# L-cysteine inhibited the growth of *Vibrio parahaemolyticus* via increasing the ROS level

**DOI:** 10.1128/aem.00097-26

**Published:** 2026-06-03

**Authors:** Qiuyan Yang, Huaipeng Fang, Liting Xu, Min Meng, Qingxi Han, Weiwei Zhang

**Affiliations:** 1 School of Marine Sciences, Ningbo University 47862, Ningbo, People's Republic of China; 2 Key Laboratory of Aquacultural Biotechnology Ministry of Education, Ningbo University 47862, Ningbo, People's Republic of China; Indiana University Bloomington 1771, Bloomington, Indiana, USA

**Keywords:** *Vibrio parahaemolyticus*, L-Cys, antibacterial effect, reactive oxygen species (ROS)

## Abstract

The emergence of multidrug-resistant *Vibrio parahaemolyticus* poses a severe threat to mariculture sustainability, highlighting the urgent need for eco-friendly antimicrobial agents. In this study, it was demonstrated that L-Cysteine (L-Cys) showed an inhibitory effect on the growth of *V. parahaemolyticus*, with a minimum inhibitory concentration of 7.5 mM. Microscopic observation and viability assay revealed that L-Cys compromised bacterial membrane integrity, ultimately leading to cell death. Transcriptomic and biochemical analyses showed that L-Cys induced the reactive oxygen species (ROS) by inhibiting terminal respiratory oxidases and key antioxidant enzymes of superoxide dismutase and glutathione reductase. Also, L-Cys disrupted metabolic processes of sulfur assimilation, one-carbon metabolism, β-oxidation, and nucleotide biosynthesis, which compromised cellular repair and survival mechanisms. The inhibition of L-Cys on *V. parahaemolyticus* was primarily attributed to the hydrogen sulfide generated by L-Cys metabolism, followed by an increase in ROS. In addition to its antibacterial effect, L-Cys also effectively reduced the bacterial swimming motility. Finally, L-Cys demonstrated broad-spectrum inhibitory effects on other pathogenic *Vibrio* spp. Our findings demonstrated that L-Cys is a promising antimicrobial agent that induces ROS-mediated membrane disruption, with the advantages of cost-effectiveness and environmental safety in the control of vibriosis.

**IMPORTANCE:**

Multidrug-resistant *Vibrio parahaemolyticus* poses a severe threat to mariculture sustainability and public health. Conventional antibiotics exacerbate antimicrobial resistance; therefore, in this study, we selected L-cysteine (L-Cys) as a potent inhibitor of *V. parahaemolyticus*, featuring cost-effectiveness, environmental safety, and ease of large-scale production. L-Cys is metabolized into hydrogen sulfide, and the latter triggers ROS burst, and it can also disrupt metabolic processes of sulfur assimilation, one-carbon metabolism, β-oxidation, and nucleotide biosynthesis. Besides, L-Cys also significantly reduced the bacterial swimming motility. Moreover, L-Cys suppresses the growth of other pathogenic *Vibrio* spp., offering a novel agent for vibriosis control.

## INTRODUCTION

Bacteria of the genus *Vibrio* are widely distributed in marine and estuarine environments (1). They can cause vibriosis, posing serious threats to aquaculture, with pathogenic isolates such as *Vibrio parahaemolyticus*, *V. alginolyticus*, *V. harveyi*, *V. anguillarum*, *V. crassostreae*, *V. splendidus*, and *V. mediterranei* frequently reported in farmed aquatic species (1, 2). These pathogens, particularly *V. parahaemolyticus*, not only lead to disease and mortality in farmed animals, but also cause human foodborne illnesses such as acute gastroenteritis and septicemia through the food chain, thereby posing significant risks to public health (1, 3).

*V. parahaemolyticus* is a halophilic gram-negative bacterium and a typical aquatic-borne pathogen, attracting considerable concern due to its ecological adaptability and strong pathogenicity (4). It infects a wide range of aquatic animals, with a wide host of nearly all major aquaculture species, including shrimp, crabs, fish, mollusks, and echinoderms (4). Its virulence factors include thermostable direct hemolysin (TDH) and TDH-related hemolysin (TRH) that disrupt host cell membrane integrity (5, 6); the Type III Secretion System (T3SS) that drives cytotoxicity and intestinal pathogenesis (6, 7); and the Type VI Secretion System subtype T6SS2 that promotes host adhesion (8). The infection of *V. parahaemolyticus* contributes to severe diseases, leading to substantial economic losses in aquaculture. For example, *V. parahaemolyticus* infection can induce Acute Hepatopancreatic Necrosis Disease (AHPND) (9) and translucent post-larvae disease (TPD) (10) in *Penaeus vannamei,* as well as hepatopancreatic tissue necrosis in *Penaeus monodon,* all of which lead to high mortality and economic loss in shrimp culture (11).

Controlling vibriosis outbreaks in aquaculture remains challenging. On one hand, elevated water temperatures (30–37°C) and increased organic matter promote *Vibrio* proliferation (12, 13). On the other hand, indiscriminate use of antibiotics has exacerbated antimicrobial resistance (14). *V. parahaemolyticus* strains exhibit resistance to multiple antibiotics, such as β-lactams and sulfonamides (14, 15). Consequently, eco-friendly agents and strategies that can control *Vibrio* populations without inducing resistance have gained attention. These include the use of probiotics such as *Bacillus* spp. and lactic acid bacteria (16), bacteriophages (17), natural plant extracts like tea polyphenols (18), and amino acid-based antimicrobials such as ε-polylysine and d-amino acid peptides (19, 20). Compared with the use of probiotics and bacteriophages, amino acid-based antibacterials offer advantages of cost-effectiveness, easily available in large amounts, low resistance risks, and environmental safety (19). Recently, L-cysteine (L-Cys) has attracted attention for its antibacterial activity, showing inhibitory effects against *Escherichia coli*, *Staphylococcus aureus*, *Listeria monocytogenes*, and *Salmonella enteritidis*, with the strongest activity against *S. aureus* (21). At a concentration of 1,000 mg/L, L-Cys significantly delays spore germination and inhibits mycelial growth of the fungus *Monilinia fructicola*, a pathogen responsible for postharvest peach brown rot (22). However, the metabolism of L-Cys showed multiple functions in bacteria. Hydrogen sulfide (H₂S) generated from intracellular L-Cys metabolism via cystathionine β-synthase (CBS) enhances *V. cholerae* colonization by promoting iron-dependent catalase KatB activity (23). Moreover, contrasting effects of H_2_S on oxidative stress response depending on the addition time in *Shewanella oneidensis* were also observed (24). However, the effect of L-Cys on the important pathogenic *Vibrio* species in aquaculture remains unexplored.

This study aims to systematically evaluate the inhibitory effect of L-Cys on *V. parahaemolyticus*. The underlying mechanisms were investigated through transcriptomic analysis, combined with assessments of cell membrane integrity, hydrogen sulfide (H_2_S) production, antioxidant enzyme activity, and intracellular reactive oxygen species (ROS) levels. Furthermore, the effect of L-Cys on the virulence factors of *V. parahaemolyticus* was examined. Finally, the inhibitory effect of L-Cys on other pathogenic *Vibrio* species was assayed. This study not only elucidates the mechanism behind the inhibition of L-Cys on *V. parahaemolyticus* but also provides a potential reagent for controlling *Vibrio* infections in aquaculture.

## MATERIALS AND METHODS

### Bacterial strain and culture

The *V. parahaemolyticus* YDE17 used in this study was obtained from our laboratory collection. The strain was routinely cultured in 2216E medium at 28°C. For carbon source screening experiments, the following media were prepared: M9 medium (Haibo, Qingdao, China) without d-glucose (Coolaber, Beijing, China); M9 minimal medium supplemented with 0.4% d-glucose as a carbon source; when solid medium was needed, 1.2% agar was added. All media were adjusted to 3% (wt/vol) salinity by adding NaCl. In liquid culture experiments, bacteria were inoculated at a ratio of 1:100 (vol/vol) and incubated at 28°C with shaking at 160 rpm.

### Screening of amino acids as the sole carbon sources

The selection of amino acids used by *V. parahaemolyticus* YDE17 was performed following the method described by Zhang et al. (25), with minor modifications. Twenty l-amino acids (Coolaber, Beijing, China) were individually supplemented as the sole carbon source in M9 agar plates. Briefly, 1 mL aliquots of bacterial culture at OD_600_ ≈ 0.5 were collected with three biological repeats, washed three times with M9 medium, and resuspended in the same medium. For liquid assays, the washed cells were inoculated into M9 medium supplemented with 40 mM of each l-amino acid. Cultures were incubated at 28°C with shaking at 160 rpm in a constant-temperature shaker (Jiecheng, Shanghai, China) for 12 h, and OD₆₀₀ was measured using a microplate reader (FlexA-200, Allsheng, Hangzhou, China). For agar plate assays, the bacterial suspensions were diluted 10³-fold, and 100 μL of each dilution was spread onto M9 agar plates containing each of the corresponding l-amino acids, with three technical replicates per sample. After incubation at 28°C for 24 h, single colonies were enumerated.

### The inhibitory effect of L-Cys on the growth of *V. parahaemolyticus* YDE17

The inhibitory effect of L-Cys was evaluated according to the method described by Wang et al. (22). Briefly, *V. parahaemolyticus* YDE17 was cultured to the exponential phase in M9 minimal medium. Cells from three independent cultures were harvested, washed three times with M9 medium, and resuspended to an OD_600_ of 0.5. Resuspended cells were then inoculated into M9 minimal medium supplemented with L-Cys at concentrations of 0, 1.25, 2.5, 5, 7.5, 10, 20, 30, 40, and 50 mM, with three biological replicates per concentration. The medium without L-Cys was used as a control. All 90 aliquots were incubated at 28°C for 24 h, and the results were photographed.

To assess growth in 2216E medium, 50 μL aliquots of *V. parahaemolyticus* YDE17 culture were inoculated into fresh 2216E media containing L-Cys concentrations ranging from 0 to 50 mM, with three biological repeats. Cultures were incubated at 28°C for 36 h. The OD_600_ was measured every 2 h during the first 12 h, and further measurements were taken at 24 and 36 h using a microplate reader.

### Scanning electron microscopy (SEM)

Samples for SEM were prepared following the method of Jahan et al. (26). *V. parahaemolyticus* YDE17 was cultured in fresh 2216E medium with or without 5 mM L-Cys, and incubated at 28°C with shaking at 160 rpm to the exponential phase. Cells were harvested by centrifugation at 3,000 ×  *g* for 10 min, fixed with 3% glutaraldehyde at 4°C for 2 h, and washed three times with 0.1 M PBS. Subsequently, samples were dehydrated through a graded ethanol series (30–100%), subjected to critical-point drying, sputter-coated with gold, and examined using a Hitachi S-3400N scanning electron microscope (Hitachi, Japan).

### Membrane integrity assays

Membrane integrity was evaluated using the LIVE/DEAD Bacterial Viability Kit (Beyotime Biotechnology, Shanghai, China) according to the manufacturer’s instructions. N,N-dimethylaniline N-oxide (DMAO), a membrane-permeable green fluorescent dye that labels all bacterial cells, and propidium iodide (PI), a red fluorescent dye that only enters cells with compromised membranes, were used. *V. parahaemolyticus* YDE17 cells at the exponential growth phase were harvested, resuspended in sterilized seawater, and divided into six aliquots. Three aliquots were treated with 5 mM L-Cys at 28°C for 30 min, then stained with the working dye solution and incubated in the dark at 37°C for 15 min. The remaining aliquots without L-Cys treatment were used as control. Finally, 10 μL of each bacterial suspension was placed onto a slide and examined under an inverted fluorescence microscope (Nikon Eclipse TI-S, Japan). Cells with intact membranes displayed green fluorescence of DMAO, while those with damaged membranes exhibited red fluorescence of PI.

### H_2_S measurement

The Lead Acetate Paper Test was conducted following the method of Ma et al. (23). Five milliliters of fresh 2216E medium was aliquoted into 15 mL glass flasks supplemented with L-Cys at final concentrations of 0, 0.125, 0.25, 0.5, 1, 1.25, 2.5, and 5 mM, respectively. Three independent replicates were prepared per concentration, thus a total of 24 samples were prepared. Each flask was inoculated with 1% (vol/vol) bacterial culture. A sterilized moistened lead acetate test strip (SSS Reagent, Shanghai, China) was fixed to the mouth of each flask using a rubber stopper. Following incubation at 28°C with shaking at 160 rpm for 12 h, color changes on the test strips were recorded.

In a parallel experiment, *V. parahaemolyticus* YDE17 cells were inoculated into 12 flasks with 2216E medium supplemented with L-Cys at concentrations of 0, 1.25, 2.5, and 5 mM, with three biological replicates per concentration. After incubation at 28°C for 12 h, the concentration of H_2_S gas in each tube was measured using an EDKORS portable H_2_S gas detector (Changzhou, China). To determine the effect of aminooxyacetic acid (AOAA), an inhibitor of pyridoxal 5′-phosphate (PLP), on the H_2_S producing enzymes of CBS/CSE (27), on H_2_S production, AOAA was added to the 2216E medium with 5 mM L-Cys, and H_2_S measurement was determined as described above.

### Bacterial motility assay

Swimming and swarming assays were performed according to the method of Li et al. (28). 2216E agar plates containing 0.25% (wt/vol) agar (for swimming motility) and 0.6% (wt/vol) agar (for swarming motility) were prepared with or without 10 mM L-Cys. For the swimming assay, 2 μL of *V. parahaemolyticus* YDE17 cells were inoculated into the center of the 0.25% agar plate. For the swarming assay, 5 μL of bacterial suspension was spotted onto the surface of the 0.6% agar plate. In each assay, three independent cultures adjusted to an OD_600_ of 0.5 were used. After static incubation at 28°C for 12 h, the radius of bacterial migration was measured.

### Real-time reverse transcriptase PCR (RT-PCR)

*V. parahaemolyticus* YDE17 was inoculated into fresh 2216E medium supplemented with 5 mM L-Cys and incubated at 28°C with shaking at 160 rpm for 24 h. A control group was cultured in parallel in 2216E medium without L-Cys. Both cultures were propagated in triplicate. Bacterial cells were harvested and washed with sterilized seawater to an OD_600_ of approximately 0.5. Total RNA was extracted using the MiniBEST Universal RNA Extraction Kit (TaKaRa, Beijing, China). Subsequently, RNA was reverse-transcribed and purified using the EasyScript All-in-One First-Strand cDNA Synthesis SuperMix for qPCR (One-Step gDNA Removal; TransGen Biotech, Beijing, China). Quantitative real-time RT-PCR was carried out on an ABI 7500 Real-Time Detection System (Applied Biosystems, USA) under the following cycling conditions: 94°C for 30 s, followed by 40 cycles of 94°C for 5 s and 60°C for 30 s. The primers used for real-time RT-PCR are listed in Table 1. Six technical replicates were included in the real-time RT-PCR. The amplification efficiencies of all primers were validated and fell within the acceptable range of 90–110%.

**TABLE 1:** Primers used for quantitative real-time RT-PCR

Primer	Sequence (5′ → 3′)	Product length
FlgPF	TGAAGATCAGCGTCTTGGCA	156 bp
FlgPR	TCACCGTAATCGCGTAGCTT	
FlgOF	TGAAAAAGTGGCTTGTCGCC	96 bp
FlgOR	GGCTGCCTGAATACGGTTCT	
FliCF1	GAAGGTGCGATGAACGAAGC	175 bp
FliCR1	GACGACGACCACCGAATGAT	
FlgKF	GTCAGAAGGTGGTAACGGCA	177 bp
FlgKR	GCGGCTTCATGCTCTAAACG	
FlgFF	GAGAACGATGCGCCAATCAC	213 bp
FlgFR	GTCTGCTTCGTAAGGCTGGT	
FliCF2	TCCATTGCACAGGTTGCTGA	121 bp
FliCR2	GGATCGCTACACGTTCTGCT	
933F	GCACAAGCGGTGGAGCATGTGG	186 bp
16SRTR1	CGTGTGTAGCCCTGGTCGTA	

### Transcriptomic sequencing and analysis

*V. parahaemolyticus* YDE17 was cultured in 2216E medium with or without 5 mM L-Cys for 24 h. Cells were harvested and adjusted to an OD_600_ of 0.5. RNA extraction, cDNA library construction, and sequencing were performed following the method of Parkhomchuk et al. (29). Total RNA was extracted using TRIzol Reagent (Thermo Fisher Scientific, USA), and RNA integrity was assessed on an Agilent 2100 Bioanalyzer (Agilent Technologies, USA). Following rRNA depletion, mRNA was purified, fragmented, and used for cDNA library construction. Libraries were prepared and sequenced on the Illumina HiSeq/MiSeq platform by Novogene (Beijing, China). The *V. parahaemolyticus* reference genome ASM19609v1 (GCF_000196095.1) was used for read alignment. Gene expression levels were quantified in FPKM using RSEM, and differentially expressed genes (DEGs) were identified with a significance threshold of *P*adj  < 0.05. GO and KEGG enrichment analyses were performed using the GOseqR package and KOBAS software, respectively (Novogene).

### ROS measurement

Intracellular ROS levels were measured using the fluorescent probe 2′,7′-dichlorodihydrofluorescein diacetate (DCFH-DA; Solarbio, Beijing, China) as described previously (30). Briefly, three independent cultures of *V. parahaemolyticus* YDE17 were grown to an OD_600_ of approximately 0.5. Cells were collected by centrifugation, washed twice with sterilized seawater, and resuspended to an OD_600_ of 0.5. Each suspension was divided into two equal aliquots: one was treated with 5 mM L-Cys for 30 min, and the other served as an untreated control. Subsequently, 1 μL of 10 mM DCFH-DA was added, followed by incubation at 37°C in the dark for 15 min. After washed, fluorescence intensity was measured using a multifunctional microplate reader (Varioskan Lux; Thermo Fisher Scientific, Shanghai, China) with excitation at 488 nm and emission at 525 nm. To confirm the role of ROS in L-Cys-induced cell death, bacterial cells were pretreated with 0.3% (wt/vol) thiourea, a ROS scavenger, prior to L-Cys exposure, and fluorescence intensity was measured after 2 h of incubation.

### Bacterial viability assay

Bacterial viability was assessed using the LIVE/DEAD Bacterial Viability Kit (Beyotime Biotechnology) according to the manufacturer’s instructions. Briefly, *V. parahaemolyticus* YDE17 was grown to an OD_600_ of 0.5, washed twice with sterilized seawater, and resuspended to the same OD_600_. Bacterial suspensions were exposed to L-Cys at concentrations of 0, 1.25, 2.5, 5, 10, and 20 mM, with three replicates per concentration, and incubated at 28°C for 30 min. To evaluate the role of ROS in bacterial cell death, 0.3% thiourea was added to parallel samples in combination with L-Cys. After stained with PI, fluorescence intensity was measured using a Varioskan Lux multimode microplate reader (Thermo Fisher Scientific, USA) at an excitation wavelength of 525 nm and an emission wavelength of 617 nm.

A standard curve was made to quantify bacterial cell mortality. *V. parahaemolyticus* YDE17 at exponential growth was divided into two groups: live cells (resuspended in sterilized seawater) and dead cells (fixed with pure ethanol at room temperature for 1 h). Both groups were washed and adjusted to an OD_600_ of 0.5. Serial mortality standards of 100%, 80%, 60%, 40%, 20%, 10%, and 0% were prepared by mixing appropriate volumes of live and dead cell suspensions, for example, 100% standard: 1 mL dead cell suspension; 80% standard: 0.8 mL dead suspension plus 0.2 mL live suspension, and so forth. Each standard sample was prepared in triplicate and measured under the same fluorescence conditions. The resulting standard curve was used to calculate mortality rates in the treatment groups.

### Measurement of redox enzyme activity and glutathione disulfide (GSSG) concentration

*V. parahaemolyticus* YDE17 was cultured with or without L-Cys, with three independent biological replicates for each treatment. Cells were harvested from each replicate, washed three times with sterilized seawater, and used for enzyme activity measurement. The activities of glutathione reductase (GR), catalase (CAT), and cytochrome c oxidase (Complex IV) were determined using commercial kits bought from Solarbio; superoxide dismutase (SOD) measurement was performed using a kit from Njjcbio (Nanjing, China). GR activity was defined as the amount of enzyme oxidizing 1 μmol NADPH per minute per 10^4^ cells at 37 °C and pH 8.0. CAT activity was expressed as the amount of enzyme degrading 1 μmol H_2_O_2_ per minute per 10^5^ cells. One unit of SOD activity (U) was defined as the amount of enzyme causing 50% inhibition in the specified assay system. One unit (U) of cytochrome c oxidase activity was defined as the amount of enzyme catalyzing the degradation of 1 nmol reduced cytochrome c per minute per mg protein.

Since GR is responsible for reducing oxidized glutathione (GSSG) to reduced glutathione (GSH), the levels of GSSG were measured to indicate whether the glutathione redox cycle was disturbed after L-Cys treatment. Intracellular GSSG content was measured using a GSH/GSSG Assay Kit (Beyotime Biotechnology) following the manufacturer’s protocol. Briefly, standard curves were generated using GSSG at different incubation time points. After the reaction was performed according to the protocol, the absorbance at 412 nm was measured. GSSG concentrations were calculated based on the standard curve.

### Effect of L-Cys on the growth of different *Vibrio* spp. strains

To assess the ubiquitous presence of L-Cys inhibition on *Vibrio* spp. strains, six strains, that is, *V. alginolyticus* H1, *V. splendidus* SSD10, *V. anguillarum* SSD9, *V. mediterranei* RPV2, *V. harveyi* W18, and *V. crassostreae* S2, were cultured in the medium supplemented with L-Cys. M9 medium supplemented with 20 mM L-Cys was first used to evaluate whether *Vibrio* spp. could utilize L-Cys as the sole carbon source. Then, L-Cys was added to the M9 minimal medium at concentrations of 0, 1.25, 2.5, 5, 10, 20, 30, 40, and 50 mM, respectively. Each concentration was tested in triplicate using 15 mL glass flasks, yielding a total of 27 experimental samples for each strain. All cultures were incubated at 28°C with continuous shaking for 12 h, after which the OD_600_ was measured.

### Statistical analysis

Data are presented as mean ± standard deviation (SD). Statistical analysis was performed using GraphPad Prism software (GraphPad Software), with group comparisons conducted by one-way analysis of variance (ANOVA) and independent samples *t*-test. Statistical significance was defined as *, *P* < 0.05, **, *P* < 0.01; the labels “*a*” and “*b*” also denote *P* < 0.05 and were used to distinguish significance levels between different comparison groups.

## RESULTS

### Amino acids showed different efficiency on the growth of *V. parahaemolyticus* YDE17

**Fig 1:**
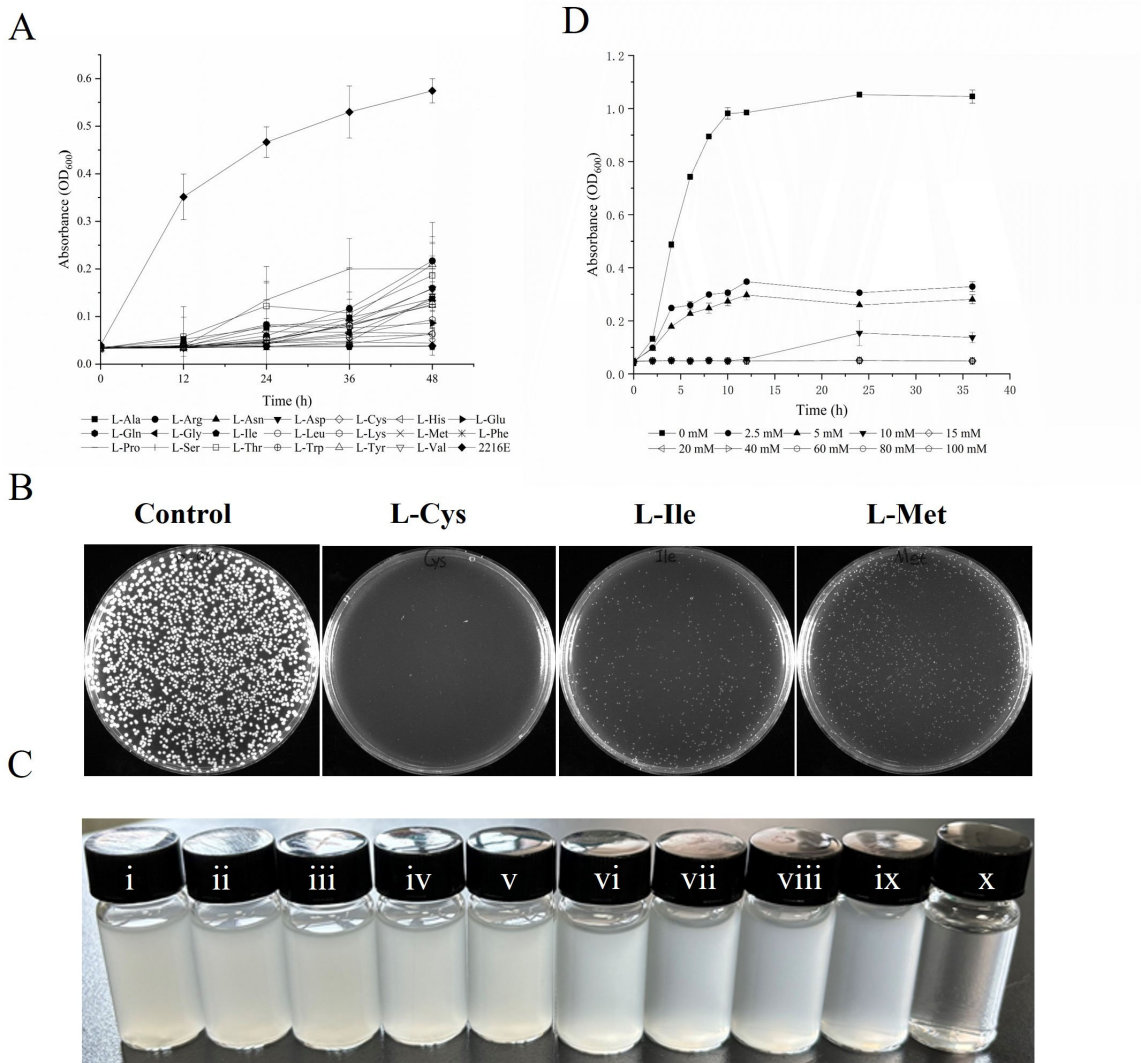
The growth of *V. parahaemolyticus* YDE17 in the presence of different amino acids. (**A**) Growth of *V. parahaemolyticus* YDE17 in M9 medium supplemented with every single amino acid as the sole carbon source. (**B**) Colony of *V. parahaemolyticus* YDE17 grown on M9 agar plates containing D-glucose (control), L-Cys, L-Ile, and L-Met as the sole carbon source, respectively. (**C**) Growth of *V. parahaemolyticus* YDE17 in M9 minimal medium supplemented with increasing concentrations of L-Cys. Symbols i–ix represent L-Cys at concentrations of 0, 1.25, 2.5, 5, 10, 20, 30, 40, and 50 mM, respectively; symbol x represents M9 medium with 20 mM L-Cys as the sole carbon source. (**D**) Growth of *V. parahaemolyticus* YDE17 in 2216E medium supplemented with different concentrations of L-Cys. The measurements were performed with three independent replicates for each concentration. Data are shown as mean ± SD.

When each amino acid was used as the sole carbon source on solid M9 agar plates, *V. parahaemolyticus* YDE17 exhibited distinct growth phenotypes, with differences in colony size and number among the 20 tested amino acids, indicating their varying efficiency as carbon sources (Fig. S1A). In liquid medium with L-Ile or L-Cys as the sole carbon source, OD_600_ remained negligible after cultured for 48 h, suggesting that these two amino acids were likely not the well-used carbon sources for *V. parahaemolyticus* YDE17 (Fig. 1A). Simultaneously, on solid medium with L-Cys as the sole carbon source, no visible colonies formed (Fig. 1B), which indicated that L-Cys could not be used by *V. parahaemolyticus* YDE17 under the tested conditions. All other amino acids supported growth to some extent; however, compared to colonies grown on 2216E plates, colony numbers were reduced when a single amino acid was used as the sole carbon source. For example, when L-Ile and L-Met were separately provided as the sole carbon source, colony counts decreased by 79% and 57%, respectively (Fig. 1B and Fig. S1B). Together, these results indicated that among all the 20 tested amino acids, L-Cys at the tested concentrations could not serve as the sole carbon source to support the growth of *V. parahaemolyticus* YDE17 like other amino acids.

### L-Cys inhibited the growth of *V. parahaemolyticus* YDE17

When different concentrations of L-Cys were added to M9 minimal medium, the OD_600_ of *V. parahaemolyticus* YDE17 declined progressively with increasing L-Cys levels. No growth was observed when 20 mM L-Cys was used as the sole carbon source (Fig. 1C). These results suggested that L-Cys exerted a dose-dependent inhibitory effect on bacterial growth under these conditions. Although the inhibitory effect was still present, L-Cys did not completely suppress growth even at 50 mM in M9 medium (Fig. 1C). To test whether the inhibition could be retained in different matrices, *V. parahaemolyticus* YDE17 was inoculated into 2216E medium supplemented with 0-50 mM L-Cys. A dose-dependent inhibition was observed within the range of 0–5 mM, leading to a gradual decrease in OD_600_ over 20 h. At concentrations above 7.5 mM, growth of *V. parahaemolyticus* YDE17 was completely inhibited (Fig. 1D).

### L-Cys damaged cellular structure and membrane integrity

**Fig 2:**
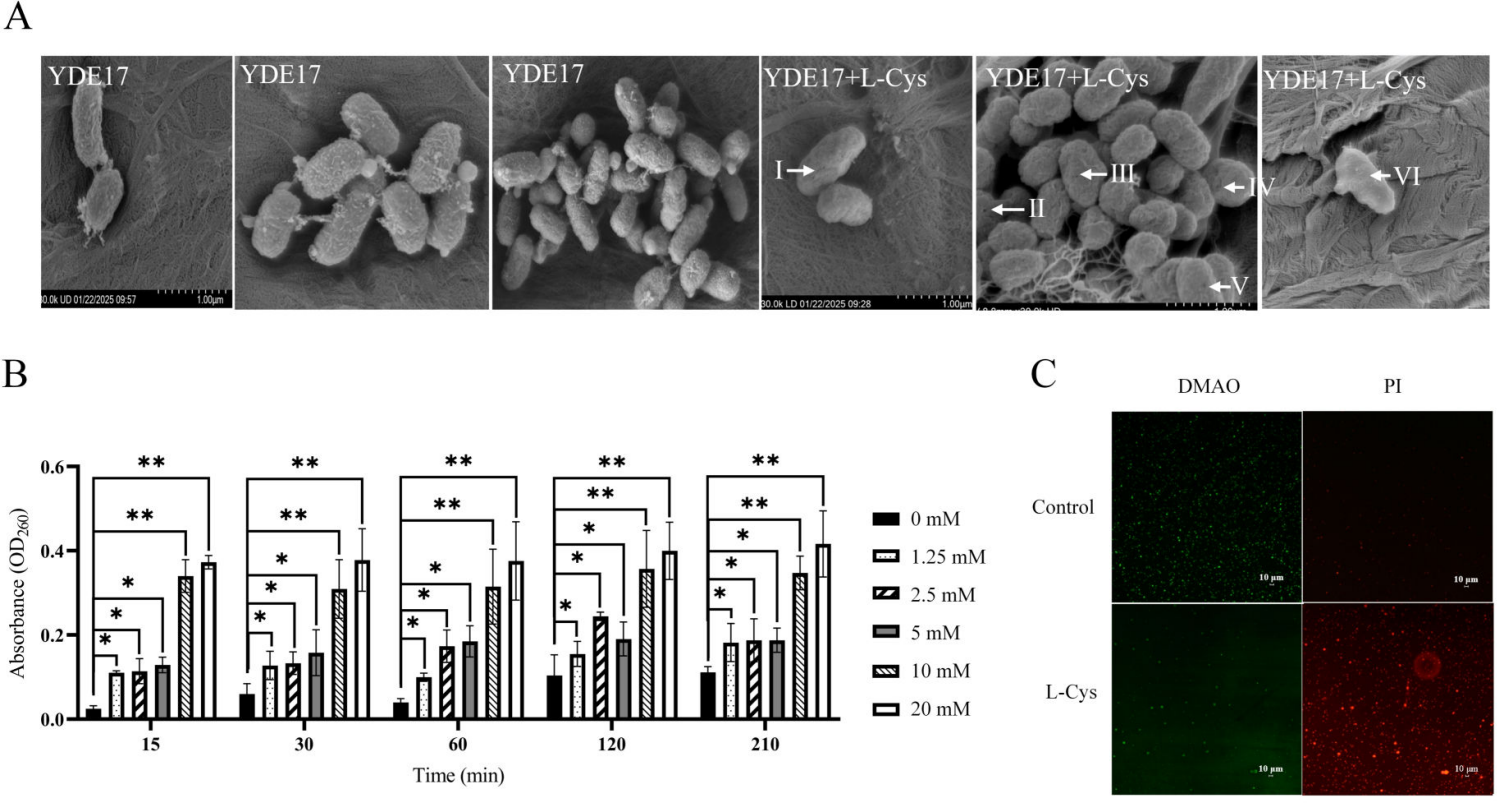
Phenotypic alterations in *V. parahaemolyticus* YDE17 cells after treatment with L-Cys. (**A**) SEM images of *V. parahaemolyticus* YDE17 cells untreated or treated with L-Cys. Arrows I–VI highlight representative regions displaying compromised cell integrity. (**B**) Dose- and time-dependent extracellular nucleic acid release from *V. parahaemolyticus* YDE17 induced by L-Cys. At each time point, the group without L-Cys was used as the control. Data are presented as mean ± SD, with at least three biological replicates per group. Statistical significance was assessed using *t*-tests, *, *P* < 0.05, **, *P* < 0.01. (**C**) Confocal laser scanning microscopy images of untreated cells and cells treated with 10 mM L-Cys. DMAO-stained green fluorescence indicates cells with intact membranes, whereas PI-stained red fluorescence labels cells with damaged membranes. Scale bar = 10 µm.

SEM showed structural alterations in *V. parahaemolyticus* YDE17 cells following L-Cys treatment. Untreated cells exhibited typical short-rod morphology, abundant filamentous extracellular secretions, and intact cell surfaces. Whereas L-Cys-treated cells displayed a reduction in filamentous accessory, accompanied by the appearance of numerous irregularly shaped and unevenly distributed pores in the cell wall (Fig. 2A). To further assess cellular damage, nucleic acid content in the culture supernatant was measured in the cells grown with increasing concentrations of L-Cys. Higher concentrations of L-Cys resulted in more nucleic acid release into the supernatant (Fig. 2B), indicating L-Cys-induced disruption of membrane integrity and leakage of cytoplasmic contents. Notably, significant cellular damage was detected within 15 min after exposure, but prolonged treatment did not intensify this effect (Fig. 2B). Bacterial viability under L-Cys exposure was evaluated using fluorescence staining. In the untreated control, DMAO-stained live cells substantially outnumbered PI-stained dead cells. However, treatment with 5 mM L-Cys markedly altered this ratio, with a significant increase in PI-positive dead cells (Fig. 2C). These results indicated that L-Cys rapidly caused severe damage to the cell membrane of *V. parahaemolyticus* YDE17, ultimately leading to bacterial death.

### Transcriptomic analysis of *V. parahaemolyticus* YDE17 in response to L-Cys

**Fig 3:**
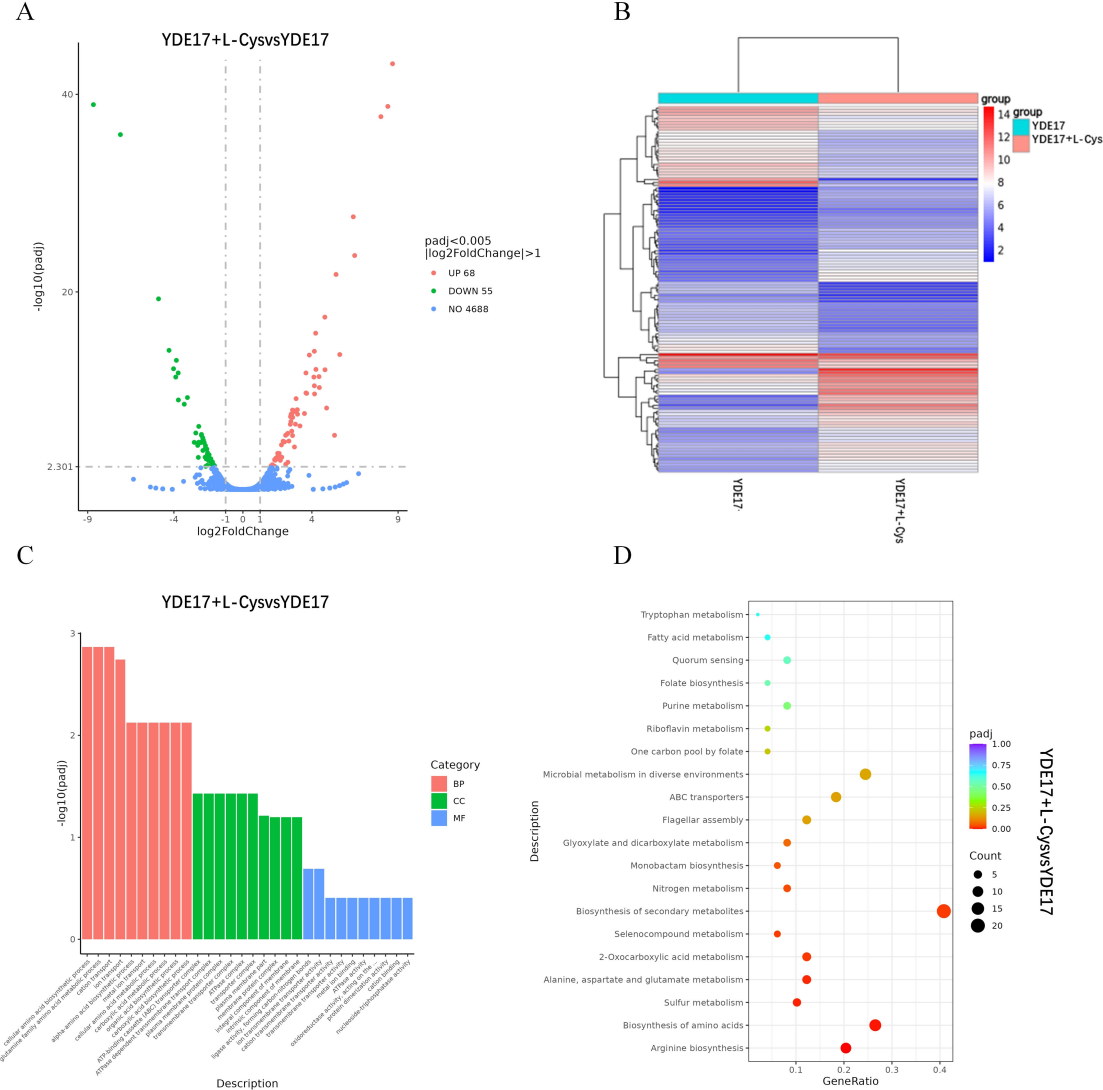
Transcriptomic analysis of *V. parahaemolyticus* YDE17 with and without L-Cys treatment. (**A**) Volcano plot of DEGs between YDE17 and YDE17 + L-Cys. The *x*-axis shows log_2_(fold change), and the *y*-axis represents −log_10_(adjusted *P* value). Red and green dots denote significantly upregulated and downregulated genes, respectively. (**B**) Hierarchical clustering of DEGs. Each row corresponds to an individual gene. Transcriptomic profiles between the two groups were visualized using MatLab. (**C**) GO enrichment analysis. The *x*-axis lists GO terms; the *y*-axis shows −log_10_(adjusted *P* value) to indicate enrichment significance. Bars represent significantly enriched terms in biological process (BP), cellular component (CC), and molecular function (MF) categories. (**D**) KEGG pathway enrichment analysis. The *x*-axis represents the ratio of DEG number to total gene number in a pathway; the *y*-axis lists pathway names. Point color and size correspond to the adjusted *P* value and the percentage of DEGs in the pathway, respectively.

Transcriptomic analysis identified 123 DEGs when compared to the expression in YDE17 + L Cys treatment groups with that in YDE17 control, comprising 68 upregulated and 55 downregulated genes (Fig. 3A). Expression levels of these DEGs were significantly altered in L-Cys-treated cells relative to the untreated control (Fig. 3B). Upregulated DEGs were primarily enriched in the following Gene Ontology (GO) categories: Biological processes (BP): localization (GO: 0051179) and amino acid metabolic processes (GO: 0008652); Cellular components (CC): membrane components (GO:0044425), ABC transporter complexes (GO:0043190), and various organelles (GO:0043226); Molecular functions (MF): enzyme activity (GO: 0016787), ion binding (GO: 0043168), and transporter activity (GO: 0022857) (Fig. 3C; Fig. S2A and Table S1). Downregulated DEGs were mainly associated with: BP: localization (GO: 0051234), ion transport (GO: 0006812), and oxidation–reduction processes (GO: 0055114); CC: organelles (GO: 0043228), cell projections (GO: 0042995), and bacterial-type flagella (GO: 0009288); MF: enzyme activity (GO: 0016491), ion binding (GO: 0043167), and transporter activity (GO: 0015075) (Fig. 3C; Fig. S2B and Table S1).

The term “Localization” (GO:0051179) emerged as the most enriched category, comprising 21 genes (42.9% of DEGs) and exhibiting bidirectional regulation. Upregulated genes were primarily associated with substrate transport and membrane-localization systems, whereas cation efflux pumps and ABC transporter subunits were downregulated, suggesting a broad activation of membrane transport functions. “Cellular amino acid biosynthetic process” (GO:0008652) showed the highest statistical significance (*P* = 1.77 × 10^−5^), with all seven related genes being upregulated, indicating its role as a central node in the amino acid metabolic network that is directly linked to the arginine biosynthesis pathway in KEGG. Seven DEGs were enriched in “oxidation–reduction process” (GO: 0055114), among which VP_RS14070, *fadE*, *VP_RS14130*, and VP_RS13375 were significantly downregulated (*P* < 0.05). Of particular note, VP_RS14070, encoding SOD, was markedly downregulated. Given its involvement in oxidation–reduction processes, metal ion binding, and antioxidant activity, the intracellular ROS level was subsequently measured in the following experiments.

KEGG pathway analysis revealed that DEGs were enriched in several metabolic and transport categories, including biosynthesis of secondary metabolites, amino acid biosynthesis, arginine biosynthesis, microbial metabolism in diverse environments, and ABC transporters (Fig. 3D and Fig. S2C and D and Table S1). Among the upregulated pathways, arginine biosynthesis (vpa00220), riboflavin metabolism (vpa00740), and fatty acid biosynthesis (vpa00061) were notably enriched. Arginine biosynthesis emerged as the most statistically significant term (*P* = 5.30 × 10^−11^) and was also the most highly enriched in both KEGG and GO analyses. This pathway contained ten genes, *argC*, *argB*, *argD*, *argF*, *argG*, *argH*, *carA*, *carB*, *astA*, and *astB*, all of which were upregulated. In contrast, pathways such as one-carbon pool by folate (vpa00670), sulfur metabolism (vpa00920), and flagellar assembly (vpa02040) were downregulated. Sulfur metabolism showed the strongest downregulation, with all five related genes, *cysI*, *cysJ*, *cysG*, *sir*, and *phsA*, being suppressed. These results indicated that L-Cys substantially altered the physiological state of *V. parahaemolyticus* by modulating multiple key metabolic and biosynthetic pathways.

### L-Cys elevated ROS through hydrogen sulfide (H_2_S) production

**Fig 4:**
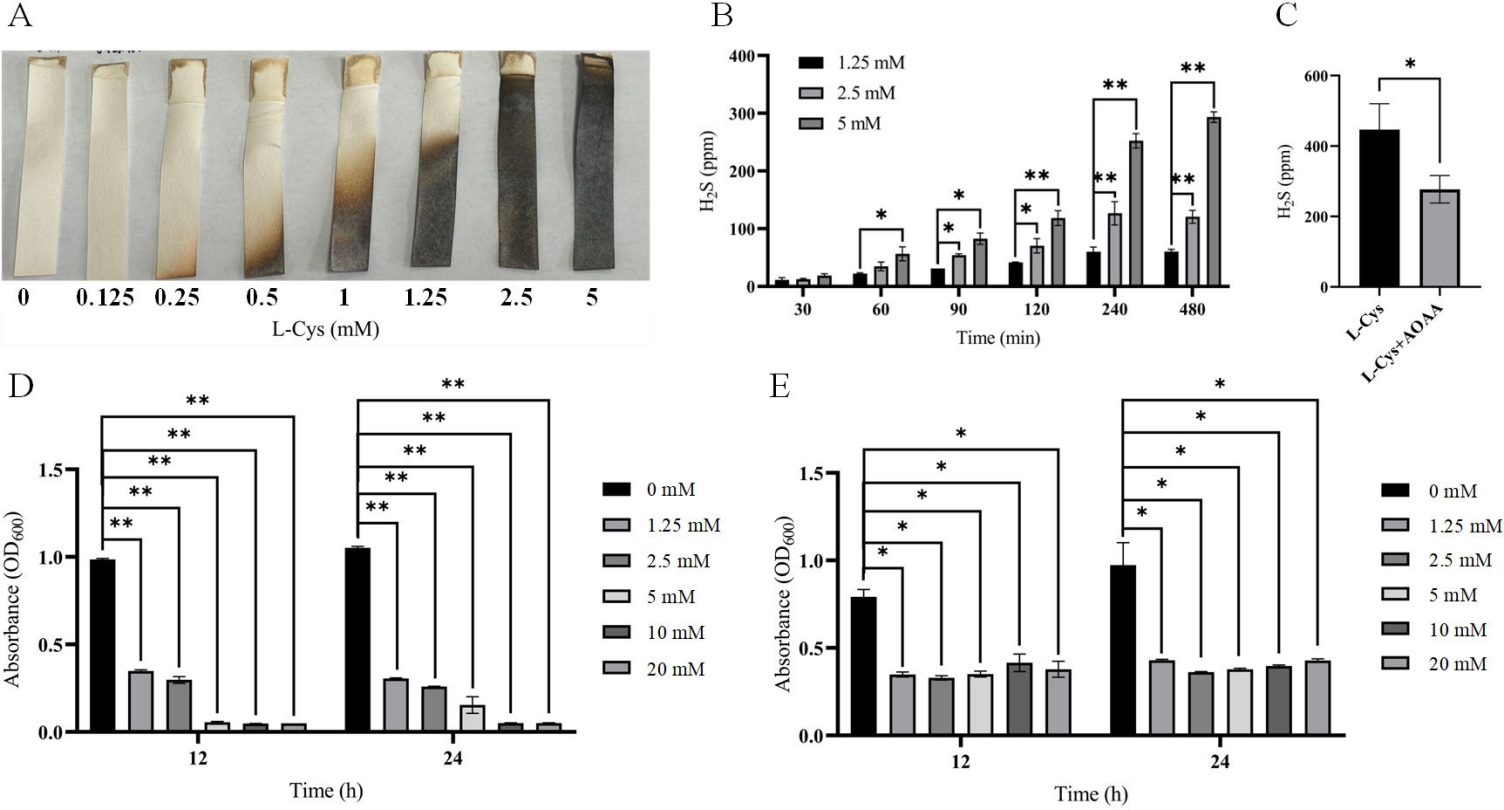
H_2_S production by *V. parahaemolyticus* YDE17 in the presence of L-Cys contributes to growth inhibition. (**A**) Qualitative detection of H_2_S using lead acetate test paper. Darker test paper with increasing L-Cys concentration indicated more H_2_S generation. (**B**) Quantitative measurement of H_2_S levels. (**C**) H_2_S production in the presence of AOAA. (**D**) OD_600_ of *V. parahaemolyticus* YDE17 in 2216E medium supplemented with L-Cys. (**E**) OD_600_ of *V. parahaemolyticus* YDE17 cultured in 2216E medium with NaHS as an exogenous H_2_S donor. For each assay, medium without L-Cys was used as a control. All data are presented as mean ± SD, with at least three biological replicates per group. Statistical significance was evaluated by *t*-test, *, *P* < 0.05, **, *P* < 0.01.

Production of H_2_S by *V. parahaemolyticus* YDE17 in the presence of exogenous L-Cys was assessed both qualitatively and quantitatively. A distinct rotten-egg odor was noted during cell growth, and the blackening of lead acetate test strips confirmed H_2_S release. The darkness of the test strips increased progressively as L-Cys concentration rose from 0 to 2.5 mM. L-Cys at concentrations above 2.5 mM, the strips turned completely black, indicating that at lower concentrations, H_2_S levels depended on the amount of exogenously supplied L-Cys (Fig. 4A). Quantitative measurements showed that after incubating for 4 h, H_2_S levels reached 60 ± 8, 126.67 ± 20.13, and 252.33 ± 12.58 ppm with 1.25, 2.5, and 5 mM L-Cys (Fig. 4B). Increasing L-Cys concentrations resulted in elevated H_2_S accumulation in a dose- and time-dependent manner. To further explore the H_2_S production, AOAA at a concentration that did not interfere with the growth was applied with L-Cys (Fig. S3). It could be seen that the level of H_2_S decreased (Fig. 4C), which indicated that the generation of H_2_S was catalyzed by the enzymes depending on PLP as the coenzymes.

**Fig 5:**
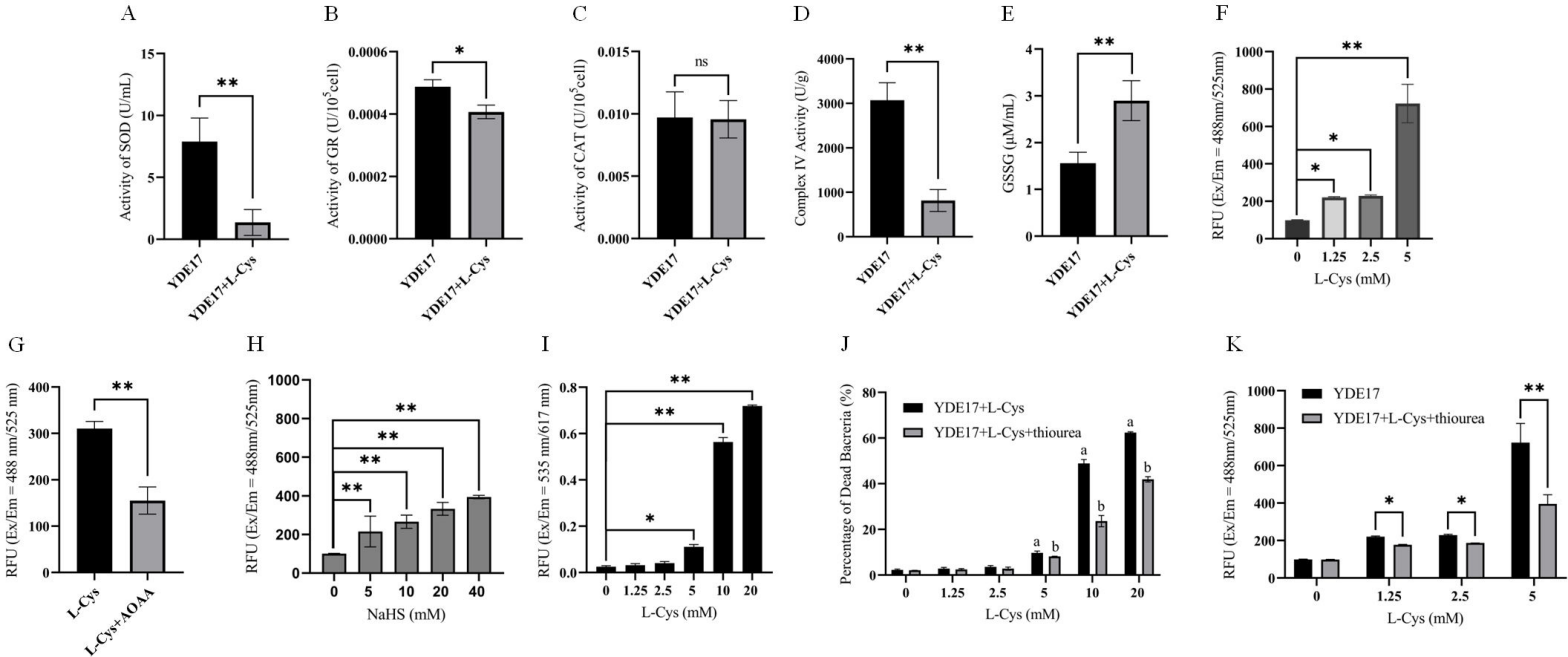
The effect of L-Cys on the antioxidant enzyme activity and oxidative stress and membrane damage in *V. parahaemolyticus* YDE17. (**A**) SOD activity, (**B**) GR activity, (**C**) CAT activity, and (**D**) cytochrome c oxidase activity of *V. parahaemolyticus* YDE17 with or without L-Cys. (**E**) Intracellular GSSG levels of *V. parahaemolyticus* YDE17 with or without L-Cys. Effect of (**F**) L-Cys, (**G**) AOAA, and (**H**) NaHS on intracellular ROS levels. (**I**) L-Cys-induced membrane permeability assessed by PI staining. (**J**) Mortality rate of *V. parahaemolyticus* YDE17 cells treated with different concentrations of L-Cys with and without thiourea. Black bars represent mortality in the “YDE17 + L-Cys” group; gray bars represent mortality in the “YDE17 + L-Cys + thiourea (0.3%)” group. Letters *a* and *b* denote significant inter-group differences (*P* < 0.05): *a* indicates comparison with the 0 mM L-Cys control; *b* indicates comparison the thiourea-supplemented group with its corresponding L-Cys group at the same concentration. (**K**) Effect of thiourea on ROS production. ROS levels after treated with L-Cys, NaHS, and thiourea were measured using a multi-mode microplate reader (Ex/Em = 488 nm/525 nm). Membrane permeability changes were assessed using the same reader (Ex/Em = 535 nm/617 nm). The group without L-Cys was used as the control in all assays. All values are expressed as mean ± SD from three independent measurements, and significance was determined by *t*-test, *, *P* < 0.05, **, *P* < 0.01.

To clarify whether L-Cys functioned primarily through H₂S, NaHS as an exogenous H_2_S donor was used added to the culture medium. The addition of NaHS also inhibited the growth of *V. parahaemolyticus* YDE17 in a dose-dependent mode, although its inhibitory effect was notably weaker than that of L-Cys at equivalent concentrations. When the concentrations of L-Cys and NaHS were below 5 mM, they showed similar inhibitory activity, with OD_600_ gradually declining as the chemical concentration increased; however, when the concentrations were higher than 10 mM, L-Cys completely suppressed growth, while the inhibitory effect of NaHS failed to achieve complete growth arrest even at higher concentrations (Fig. 4D and E). Based on transcriptomic analysis, one gene encoding the antioxidant defense enzyme SOD was significantly downregulated. To comprehensively explore the effect of L-Cys on redox homeostasis, the enzyme activities of representative redox homeostasis-related enzymes, SOD, CAT, GR, and cytochrome c oxidase were all determined. After treated with 5 mM L-Cys, SOD activity significantly decreased by 82.71% (Fig. 5A), which was highly consistent with the transcriptomic results; GR activity was significantly reduced by 16.51% (Fig. 5B), while CAT activity showed no obvious change (Fig. 5C). The enzyme activity of cytochrome c oxidase decreased significantly to 814.07 U/g after L-Cys treatment, with a reduction of 73.5% compared with the control group (Fig. 5D). Since GR is the key rate-limiting enzyme mediating the balance between GSSG and GSH in the glutathione cycle, GSSG content was simultaneously measured to verify the disturbance of the glutathione cycle. After treated with L-Cys, GSSG content in *V. parahaemolyticus* YDE17 increased from 1.561 to 2.898 μM, with an increase of 85.6% (Fig. 5E and Fig. S4).

Based on the above results, the intracellular ROS levels in *V. parahaemolyticus* YDE17 in the presence of L-Cys were measured. As shown in Fig. 5F, ROS fluorescence intensity increased from 98.8 RFU in untreated cells to 220.2 ± 4.10 , 228.6 ± 5.52 , and 722.6 ± 102.89  RFU in the presence of 1.25, 2.5, and 5 mM L-Cys, demonstrating that L-Cys significantly induced ROS production. The addition of AOAA also led to a reduction of the ROS by 50% (Fig. 5G). Parallel experiments using NaHS as an H_2_S donor were performed. ROS fluorescence intensity in the presence of 10 mM NaHS reached only 265.8 ± 33.7 RFU, which was markedly lower than that 722.6 ± 102.89 RFU observed using the same concentration of L-Cys (Fig. 5H). These results suggested that H_2_S generated from L-Cys attributed to the elevated ROS levels, but there might exist other pathways, that is, the metabolic and biosynthetic pathways as determined using transcriptomic analysis.

Further membrane integrity assessment via PI staining showed that L-Cys concentrations above 5 mM significantly increased membrane permeability in *V. parahaemolyticus* YDE17 (Fig. 5I). Based on a bacterial viability standard curve (Fig. S5), mortality rates reached approximately 9.68 ± 0.83%, 48.91 ± 1.64%, and 62.36 ± 0.40% in groups treated with 5, 10, and 20 mM L-Cys, respectively (Fig. 5J). To determine whether ROS directly mediated bacterial death, the ROS scavenger thiourea at 0.3% (wt/vol) was applied into the medium, a concentration chosen via minimum inhibitory concentration (MIC) assays against *V. parahaemolyticus* YDE17 (Fig. S6). This concentration was selected for its non-growth-inhibition to ensure the reliability of ROS scavenging activity. PI staining revealed that thiourea markedly enhanced bacterial survival under L-Cys treatment (Fig. 5I). Subsequently, ROS measurement showed that thiourea significantly attenuated ROS levels induced by L-Cys (Fig. 5K). This result confirmed that the ROS inhibitor thiourea reduced both intracellular ROS and mortality of *V. parahaemolyticus* YDE17, supporting that excessive ROS accumulation was the agent leading to irreversible cell death.

### L-Cys inhibited the swimming motility of *V. parahaemolyticus* YDE17

**Fig 6:**
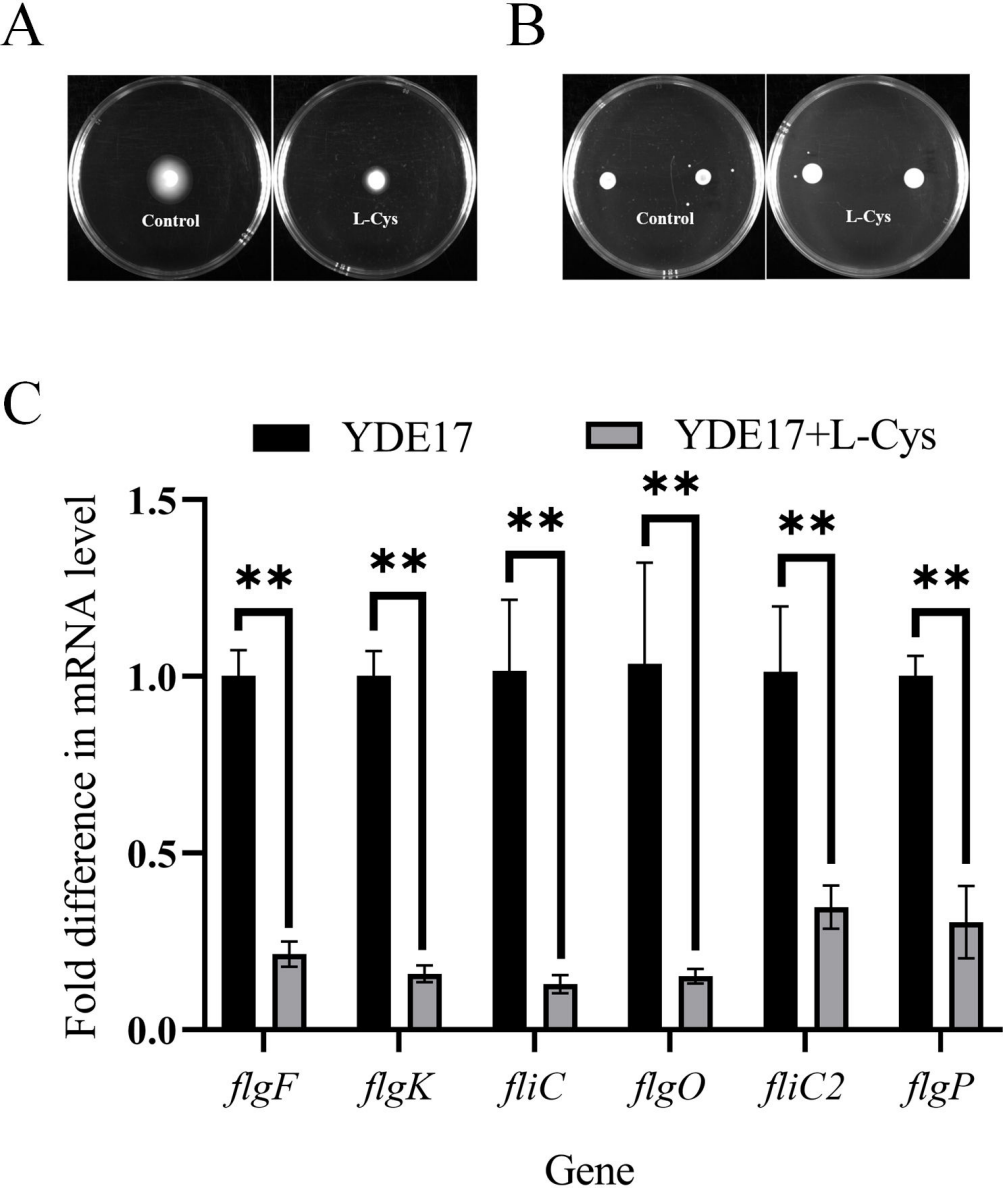
The inhibitory effect of L-Cys on the bacterial motility of *V. parahaemolyticus* YDE17. (**A**) Swimming motility in 2216E soft agar. Control group: 0 mM L-Cys; treatment group: 10 mM L-Cys. Plates were inserted with 2 µL of bacterial suspension and incubated statically at 28°C for 12 h. (**B**) Swarming motility in 2216E semi-solid agar. Control: 0 mM L-Cys; treatment: 10 mM L-Cys. Plates were dropped with 5 µL of bacterial suspension and incubated statically at 28°C for 12 h. (**C**) qRT-PCR analysis of flagellar assembly-related genes identified via transcriptomic analysis. Gene expression levels in L-Cys-treated cells were compared to that of the untreated control. Data represent three independent experiments; significance was assessed by *t*-test, **P < 0.01.

The global regulation of L-Cys led us to wonder whether L-Cys influenced virulence-associated characteristics of *V. parahaemolyticus* YDE17. In swimming assays, untreated bacteria formed an approximately spherical migration zone with a diameter of 2.07 ± 0.27 cm, with cell density gradually decreasing from the center outward. In contrast, in the presence of 5 mM L-Cys, migration was largely confined near the inoculation site, resulting in a significantly smaller migration zone (1.32 ± 0.08 cm; Fig. 6A), indicating that L-Cys inhibited the swimming motility of *V. parahaemolyticus* YDE17. For swarming motility, the control group formed a migration zone of approximately 0.7 ± 0.01 cm in diameter, and the L-Cys-treated group showed a slightly larger zone of 0.9 ± 0.03 cm. The difference was not statistically significant, suggesting that L-Cys did not substantially affect swarming motility (Fig. 6B). These phenotypic observations aligned with transcriptomic data, which showed that genes involved in flagellar assembly, including *flgO*, *flgP*, *flgK*, *flgF*, *fliC*, and *fliC2*, were downregulated after 5 mM L-Cys treatment. This downregulation was further confirmed by real-time RT-PCR (Fig. 6C). The reduced expression of these flagellar-related genes likely impaired the function of key flagellar structures, such as the H ring, rod, L/P ring, hook-filament junction, and filament, thereby reducing bacterial motility.

### L-Cys exhibited universal inhibitory effects on the growth of *Vibrio* spp.

We further investigated whether L-Cys exhibited broad-spectrum inhibitory effects on the growth of diverse marine *Vibrio* species. It is uniform that when L-Cys was used as the sole carbon source under the tested conditions, none of the tested *Vibrio* spp. strains showed obvious growth (Fig. S7 and group I). The growth of all six strains was inhibited in the presence of L-Cys in M9 minimal medium. Notably, *V. anguillarum* SSD9 was completely inhibited by L-Cys, whereas *V. alginolyticus* H1, *V. splendidus* SSD10, *V. mediterranei* RPV2, *V. harveyi* W18, and *V. crassostreae* S2 exhibited a dose-dependent inhibition pattern similar to that observed in *V. parahaemolyticus* YDE17, with progressively stronger suppression as L-Cys concentration increased (Fig. S7).

## DISCUSSION

In this study, we demonstrated for the first time that *V. parahaemolyticus* was highly sensitive to L-Cys and L-Cys was unable to be used as the sole carbon source. Addition of L-Cys into the culture medium significantly inhibited the growth of *V. parahaemolyticus* YDE17 in a dose-dependent manner. This growth suppression was directly linked to the disruption of cell membrane integrity, consistent with the previously reported antibacterial activity of L-Cys against *E. coli* (21, 31), *S. aureus* (21), and *Monilinia fructicola* (22). Notably, L-Cys exhibits a dual, concentration-dependent effect in *E. coli*: it serves as a sulfur source and reducing agent that alleviates oxidative stress and promotes growth at concentrations lower than 1 mM (32, 33); however, it inhibits growth through metabolic interference at concentrations higher than 5 mM (34). However, such a dual effect was not observed in *V. parahaemolyticus* YDE17 in our present study. This divergence may be due to the relatively high L-Cys concentrations or intrinsic strain-specific traits. Moreover, L-Cys at concentrations above 7.5 mM completely inhibited the growth of *V. parahaemolyticus* YDE17 in 2216E medium, whereas even 100 mM L-Cys did not achieve complete inhibition in M9 minimal medium. This finding suggested that the impact of exogenous L-Cys on bacteria is not only concentration- and species-dependent, but also strongly influenced by the nutritional composition of the medium. This phenomenon may be closely related to microbial metabolic networks, antioxidant capacity, and L-Cys transport efficiency (21), as similarly reported in fungal pathogens (22). Previous studies indicate that L-Cys exerts antibacterial effects through multiple pathways, including H_2_S production that disrupts the electron transport chain and ROS balance, followed by oxidative damage (30, 35), or metabolic interference-driven growth inhibition (36), and it also showed synergistic bactericidal effects with hydrogen peroxide (37).

Transcriptomic analysis and the key parameters were combined to explore the inhibition mechanism, and it showed that when L-Cys was added, DEGs were primarily enriched in processes including substance localization, amino acid metabolism, ion transport, and redox reactions. Among them, three key expression shifts synergistically drove the observed ROS burst: First, downregulation of the gene VP_RS1407 encoding SOD and gene VP_RS00355 encoding GR, as well as their activities, directly compromised the bacterial ROS-scavenging capacity, similar to the previously reported decreases in SOD activity in *E. coli* (38). SOD eliminates superoxide anions, and GR sustains GSH regeneration. Decrease in the enzymatic activity of these enzymes leads to GSH depletion, weakens ROS-scavenging capacity, and interferes with redox homeostasis. These findings are in agreement with earlier reports that L-Cys promoted ROS accumulation in *E. coli* B2 (36), and that the increase in ROS level was due to the inhibitory effect of H₂S on SOD and GR activities (30). On the other hand, the upregulation of *ribA* (VP_RS09335), encoding a rate-limiting enzyme in riboflavin synthesis, likely led to accumulation of flavin mononucleotide (FMN) and flavin adenine dinucleotide (FAD), which can promote electron leakage from the respiratory chain and further stimulate ROS production (39). Besides, according to the study in *S. oneidensis*, H_2_S targeted and inhibited the activity of complex IV, a typical heme-copper oxidase and a pivotal enzyme responsible for respiratory chain terminal oxidation (40). The inactivation of complex IV directly blocks the final step of electron transfer, forcing electrons to leak from upstream complexes followed by reaction with O_2_ to form O₂⁻, ultimately triggering excessive intracellular ROS accumulation (41). Additionally, suppression of redox-related processes (GO: 0055114) and sulfur metabolism (vpa00920) jointly disrupted the antioxidant defense system, providing molecular support for the ROS-mediated bacterial death (42). It thus can be concluded that an intracellular ROS burst might have contributed to oxidative damage and bacterial death.

In the present study, we have verified that a large amount of H_2_S was detected during *V. parahaemolyticus* in the presence of L-Cys, in which the enzymes of CBS and CSE (PLP-dependent cysteine lyases) might be involved in this process deduced from the reduced H_2_S in the presence of AOAA (43). The H_2_S produced via L-Cys metabolism contributed to the death of *V. parahaemolyticus* to a large extent, which was similar to *E. coli* (30). The increased ROS level well supported the transcriptomic analysis, which was similar to that in *E. coli* (44). Combined with the above transcriptomic analysis and the measurements of ROS under the supplement of L-Cys, AOAA, and NaHS indicated that the toxicity of L-Cys is attributed to the direct inhibition of key respiratory oxidases that block the respiratory electron transport chain and disrupt microbial energy metabolism (35), and it also suppresses the activity of antioxidant enzymes that promote the accumulation of intracellular ROS, thereby amplifying oxidative damage to microbial cells (45). This is consistent with previous reports that elevated H_2_S can inhibit antioxidant enzyme activity (22), disrupt the electron transport chain (6), and induce ROS accumulation to suppress microbial growth (45).

L-Cys broadly suppresses central metabolic and repairing pathways in the bacteria. The upregulation of arginine might first play important roles in scavenging intracellular ROS by participating in the synthesis of reduced thiol pools such as mycothiol and ergothioneine (46); however, the persistent upregulation of arginine biosynthesis might competitively consume the reducing power required for the GSH cycle, reducing the ROS scavenging efficiency (47); Eventually, arginine could promote NO production that reacts with superoxide anions to form ONOO⁻, further aggravating oxidative stress (48). The significant downregulation of the sulfur assimilation genes represented a negative feedback adaptation of *V. parahaemolyticus* to high exogenous L-Cys levels. As extracellular L-Cys adequately fulfills cellular sulfur-containing amino acid requirements, the bacterium represses these key genes to minimize metabolic redundancy (49). Downregulation of *fadE* (*VP_RS11110*)*,* a key enzyme in fatty acid β-oxidation, disrupted lipid metabolism and energy production (50). Downregulation of *fhuF* (VP_RS19860), a gene involved in iron-sulfur cluster homeostasis (51), which may disrupt cluster balance and exacerbate oxidative damage. Together, reduced expression of assimilatory sulfite reductase (VPA0803), glycine cleavage system T-protein (VPA0805), and ribonucleotide reductase (VPRS09190) impaired one-carbon metabolism, serine metabolism, and nucleotide synthesis. This extensive inhibition compromised the synthesis and repair of proteins, lipids, nucleotides, and DNA, thereby depriving cells of the capacity to counteract oxidative damage (52, 53). The changed gene expression profiles in redox, metabolic, and energetic balance also explained why L-Cys exhibited substantially stronger antibacterial activity than the same concentration of NaHS, a pure H₂S donor.

The reduction in swimming motility of *V. parahaemolyticus* YDE17 supported the metabolic regulation of virulence, a phenomenon also noted in *Cryptococcus neoformans*, in which overexpression of the RNA-binding protein Puf4 reprogrammed carbon metabolism and elevated ROS levels, significantly decreasing the synthesis of virulence factors such as capsule and melanin (54). Notably, we extended the L-Cys inhibition to diverse pathogenic *Vibrio* species. L-Cys suppressed the growth of all tested *Vibrio* strains, indicating that L-Cys possesses a broad-spectrum inhibitory effect on pathogenic *Vibrio* spp. Collectively, the multifaceted and wide inhibitory spectrum of L-Cys provides a promising basis for the development of strategies to control infection of *Vibrio* spp., especially *V. parahaemolyticus*.

### Conclusion

**Fig 7:**
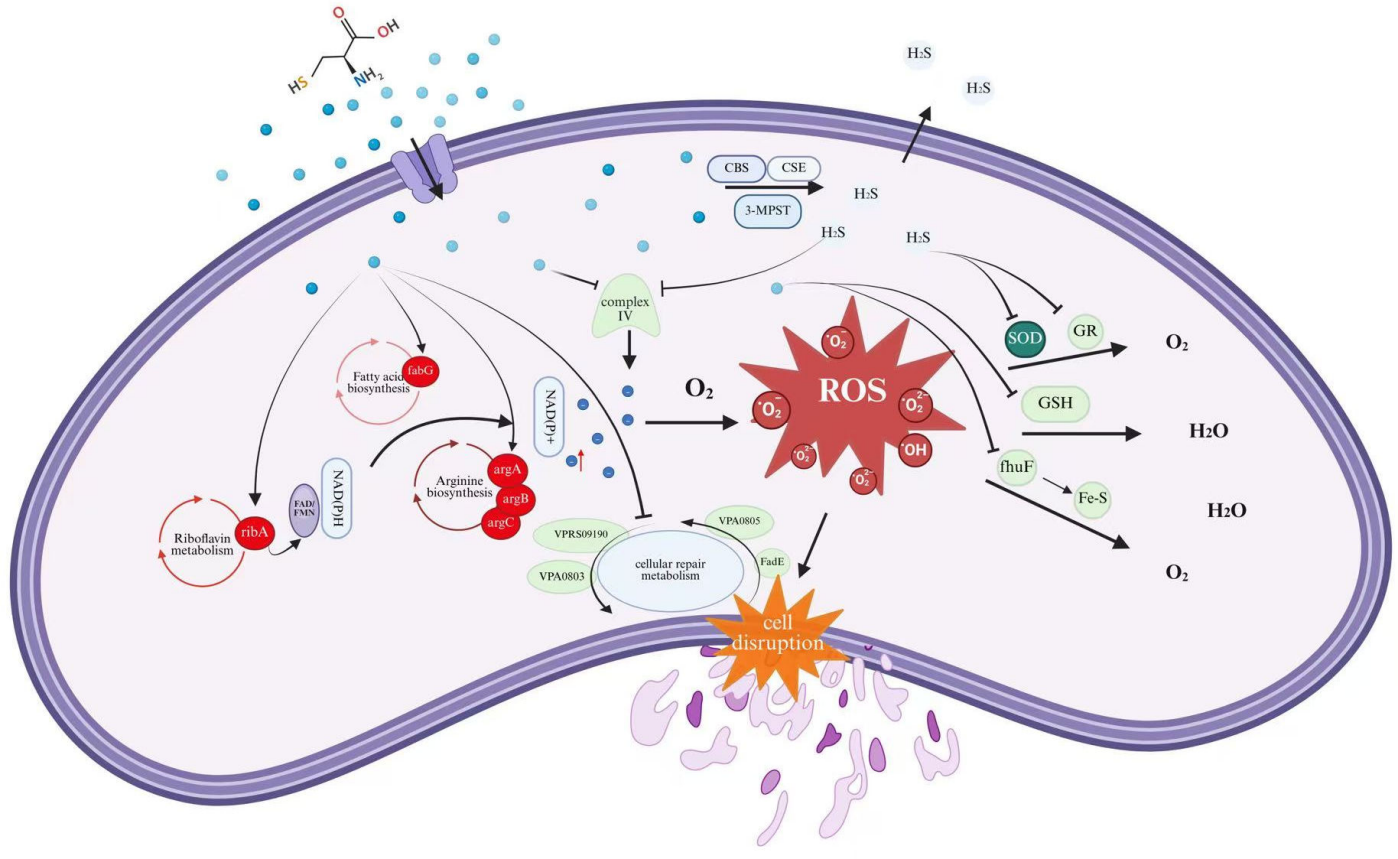
Schematic diagram showing the biological processes that are involved in the L-Cys-mediated cell death of *V. parahaemolyticus* YDE17.

This study clearly showed that L-Cys had an inhibitory effect on the growth of *V. parahaemolyticus*. The mechanism is initiated by the intracellular metabolism of exogenous L-Cys, leading to a high endogenous accumulation of H₂S. Functioning as the core effector, H₂S orchestrates a catastrophic oxidative cascade by dually targeting cellular redox homeostasis: it directly inhibits terminal respiratory oxidases (e.g., cytochrome c oxidase), thereby promoting ROS generation, while concurrently suppressing key antioxidant enzymes like superoxide dismutase and glutathione reductase, which cripples the ROS-scavenging capacity. This increased production and decreased clearance synergy results in an overwhelming ROS burst. Concurrently, L-Cys and/or its metabolites disrupt central metabolic processes, including arginine biosynthesis, sulfur assimilation, and nucleotide biosynthesis, which compromise cellular repair and survival mechanisms (Fig. 7). The broad inhibitory activity of L-Cys on other *Vibrio* species underscores its potential as a wide-spectrum antibacterial agent, offering a novel strategy for controlling vibriosis in aquaculture.

## Data Availability

The raw sequencing data have been deposited in the NCBI SRA database under accession number PRJNA1394195. The data presented in this study are available from the corresponding author upon reasonable request.

## SUPPLEMENTAL MATERIAL

Supplemental figures aem.00097-26-s0001.docx Fig. S1 to S7.

Table S1 aem.00097-26-s0002.xlsx DEGs between the YDE17+L-Cys treatment groups and the YDE17 control.

ASM does not own the copyrights to Supplemental Material that may be linked to, or accessed through, an article. The authors have granted ASM a non-exclusive, world-wide license to publish the Supplemental Material files. Please contact the corresponding author directly for reuse.
