# Supplementary material for: L-cysteine inhibited the growth of Vibrio parahaemolyticus via increasing the ROS level
Source: Appl Environ Microbiol. 2026 Jun 3;92(7):e00097-26. doi: 10.1128/aem.00097-26 (PMC13390350; doi:10.1128/aem.00097-26)
Supplement: Supplemental figures — Fig. S1 to S7. [file aem.00097-26-s0001.docx]

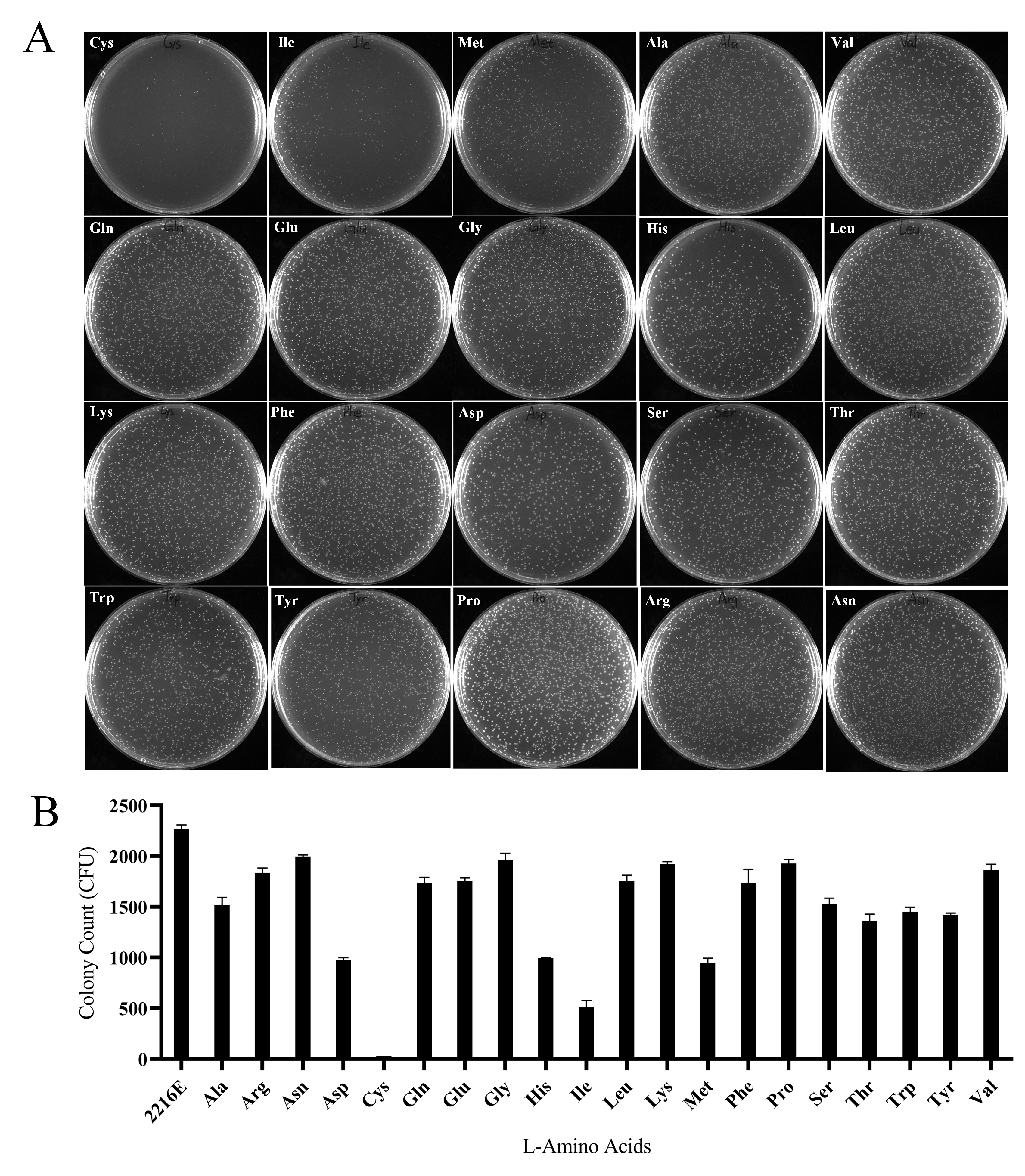


**Fig. S1** Use of each amino acid as sole carbon source by *V. parahaemolyticus*YDE17. (A) The growth of *V. parahaemolyticus* YDE17 on M9 medium agar plate supplemented with each L-amino acid as the sole carbon source, and one representative photograph of each plates were given. (B) Quantitative assessment of colony number of plate count for each amino acid as shown in (A). The data was given as means ± SD.


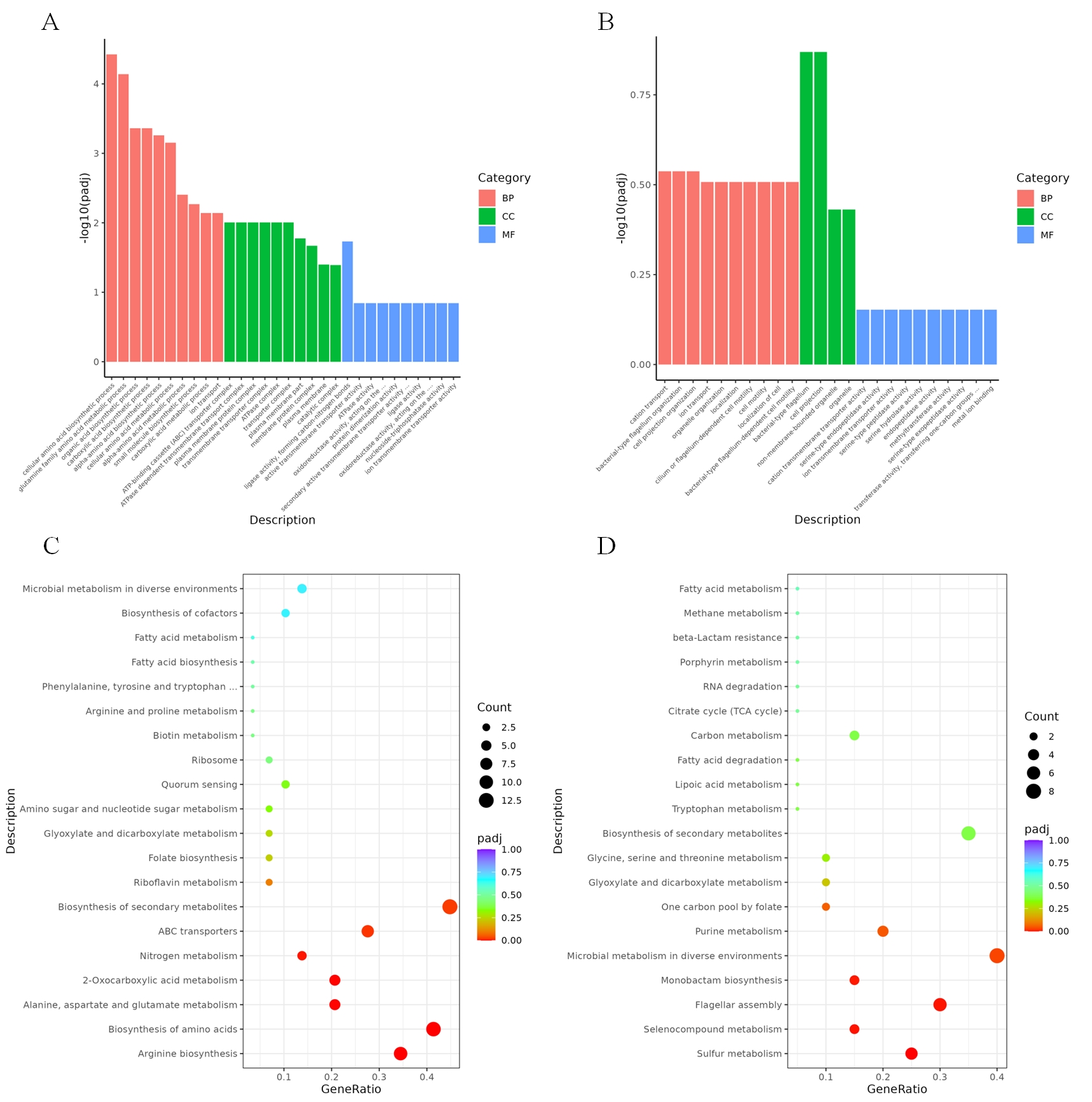


**Fig. S2** GO and KEGG enrichment analyses of DEG. (A) GO enrichment histogram for upregulated genes. (B) GO enrichment histogram for downregulated genes. (C) KEGG enrichment scatter plot for upregulated genes. (D) KEGG enrichment scatter plot for downregulated genes. In the GO plots, the x‑axis represents GO terms grouped into BP, CC, and MF; the y‑axis shows −log₁₀(adjusted *P*‑value), with bar height reflecting enrichment significance. In the KEGG plots, the x‑axis indicates the ratio of DEG number to total gene number in the pathway; the y‑axis lists pathway names. Dot color corresponds to the adjusted P‑value (darker indicates higher significance), and dot size represents the proportion of DEGs in the pathway.


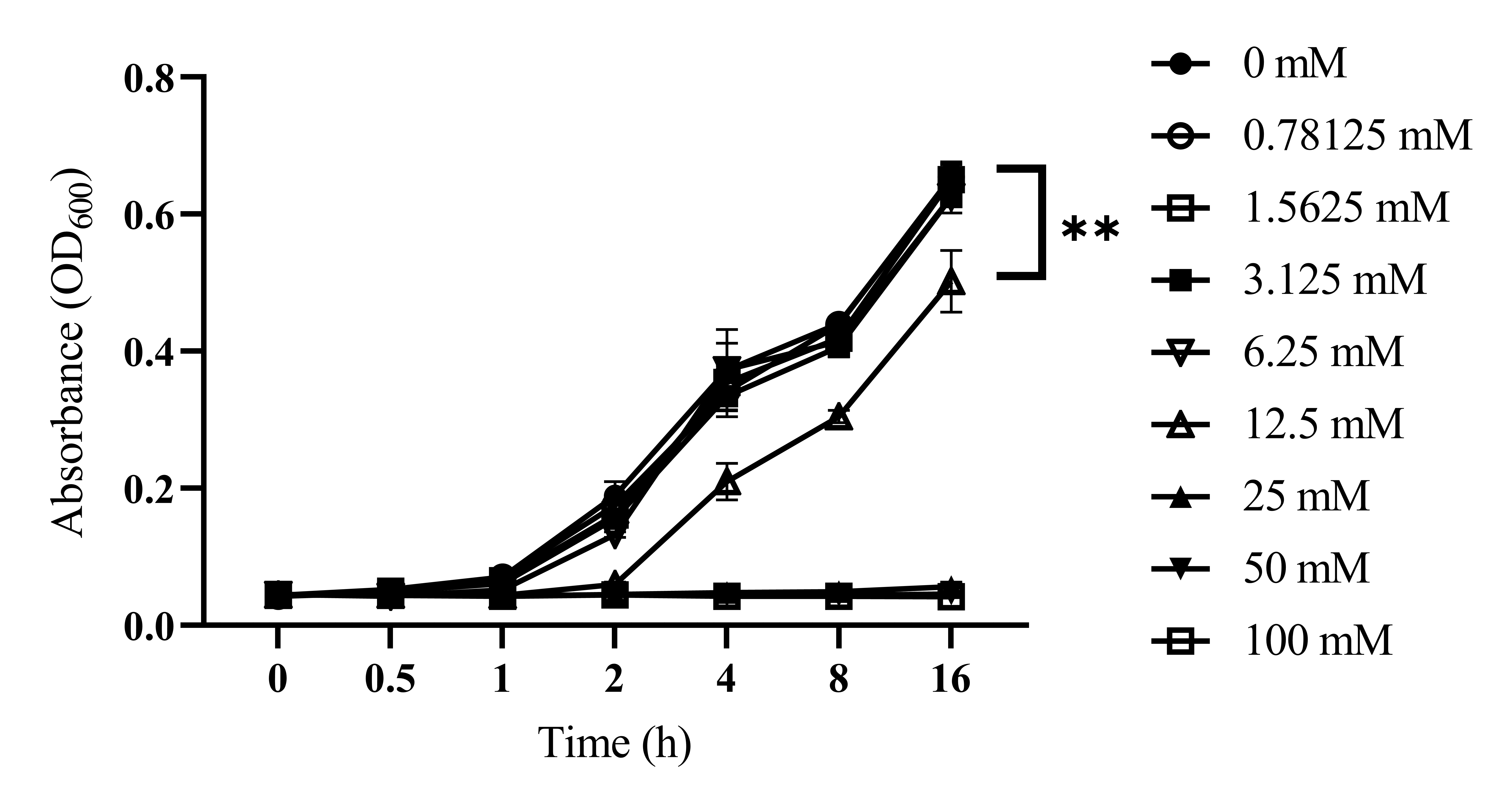


**Fig. S3** The growth of *V. parahaemolyticus* YDE17 under different concentrations of AOAA. Bacterial growth was determined by OD₆₀₀, and all data were presented as the mean ± SD from three independent experiments. ** indicated an extremely significant difference (P < 0.01) in biomass between the 12.5 mM AOAA group and the 0 mM control group at 16 h of incubation. The growth of *V. parahaemolyticus* YDE17 was completely inhibited when the AOAA concentration was ≥ 25 mM, while 6.25 mM was the highest AOAA concentration that exerted no influence on the growth of strain YDE17.


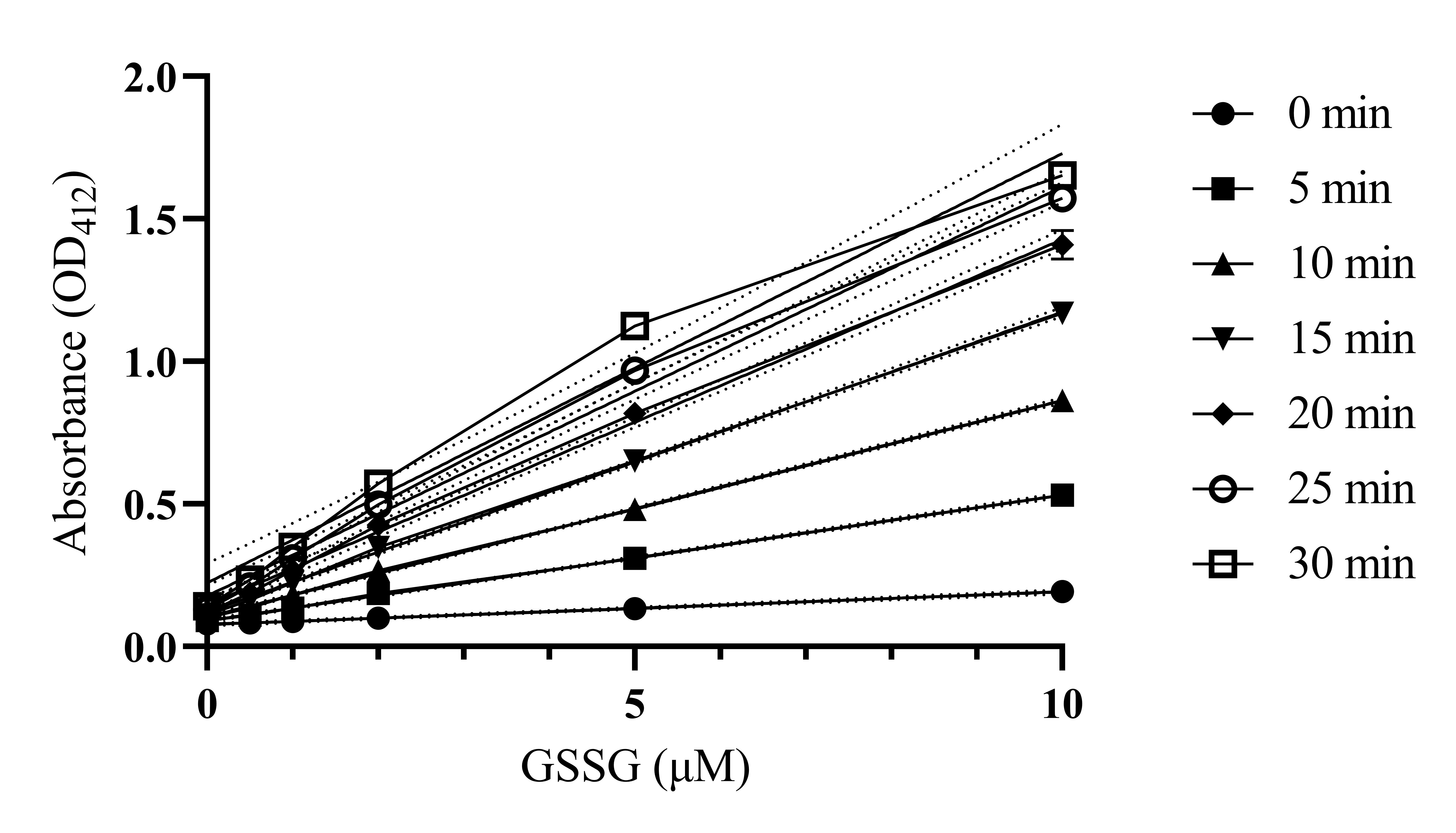


**Fig. S4** GSSG standard curves measured at different incubation time points. Data from the 25 min time point were selected for quantification (linear equation: Y = 0.1435X + 0.1774, R² = 0.9918).

**
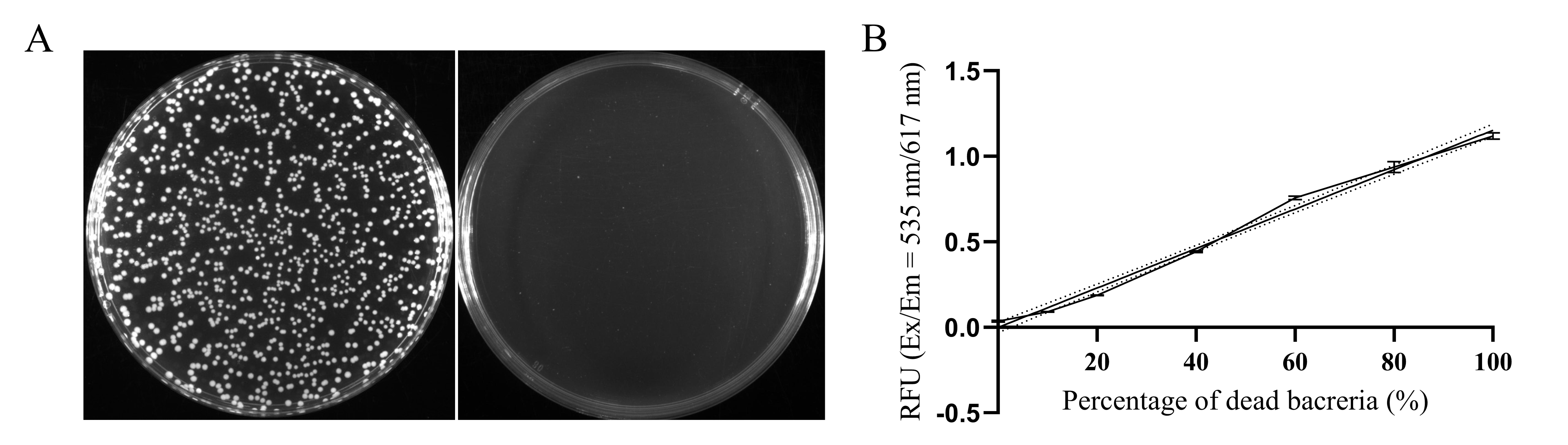
**

**Fig. S5** (A) Validation of viable bacterial cell number after ethanol treatment. Left panel: cells before ethanol treatment. Right panel: cells after 1 h of ethanol treatment. (B) Mortality standard curve for *V. parahaemolyticus* YDE17. Linear equation: Y = 0.01154X − 0.0007625, R² = 0.9903. The x‑axis shows percent of dead bacteria; the y‑axis indicates corresponding PI fluorescence intensity.


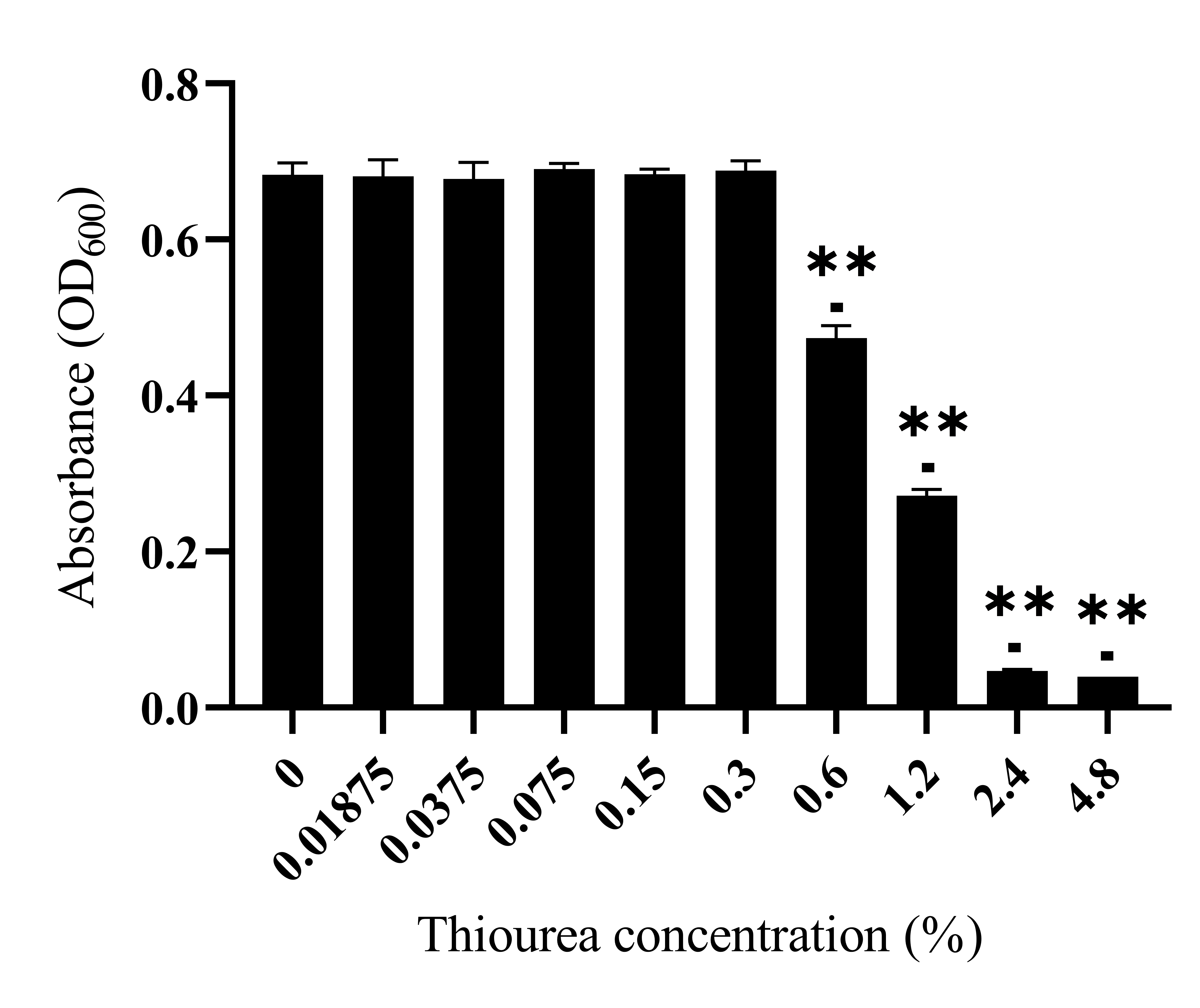


**Fig. S6** The effect of thiourea on the growth of *V. parahaemolyticus* YDE17. Bacterial cells was cultured in 2216E medium with different concentrations of thiourea for 12 h, and OD_600_ was measured. The medium without thiourea was used as a control. The data was given as means ± SD. ** meant *P*<0.05 with significance difference.


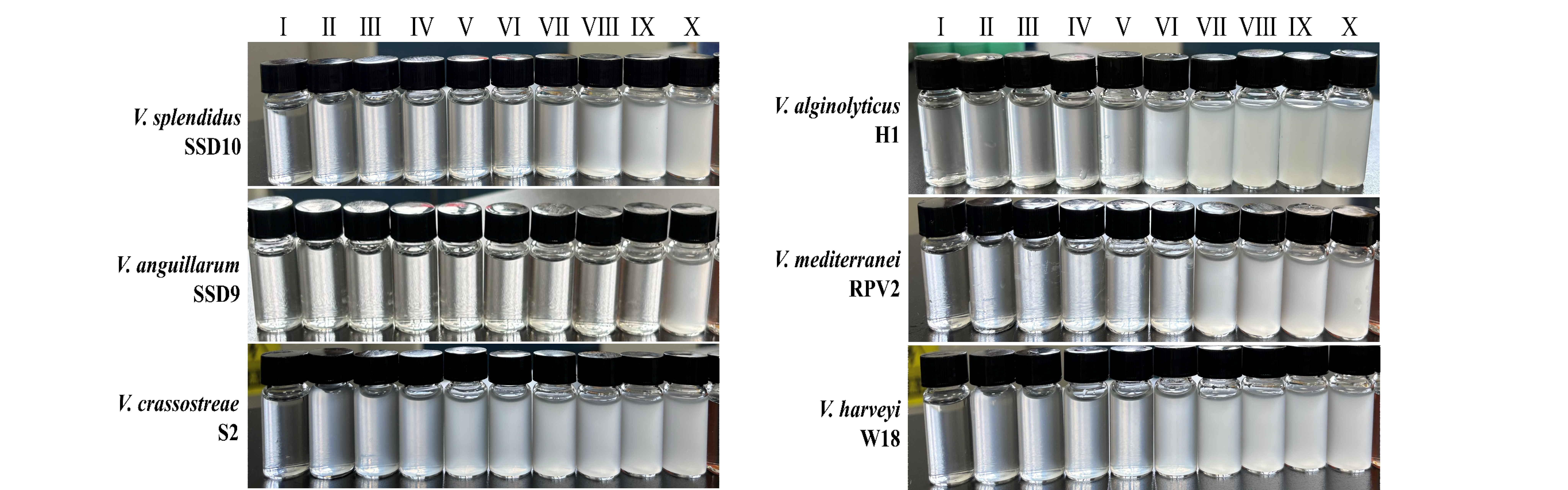


**Fig. S7** Growth inhibition of different *Vibrio* spp. by L‑Cys. Group Ⅰ: M9 medium supplemented with 20 mM L‑Cys before bacterial inoculation. Groups Ⅱ–Ⅹ: M9 minimal medium supplemented with 50, 40, 30, 20, 10, 5, 2.5, 1.25, and 0 mM L‑Cys, respectively, followed by inoculation with the tested strains. Growth inhibition was visually observed and recorded by photography.
